# Implementing clinical guidelines for gestational weight gain care: a novel application of best–worst scaling to prioritise barriers

**DOI:** 10.1186/s12913-025-13108-7

**Published:** 2025-07-29

**Authors:** Eva Farragher, Laura A. Wall, Olivia Wynne, John Wiggers, Jenna Hollis, Luke Wolfenden, Francesco Paolucci, Justine Daly, Carly Mallise, John Attia, Craig Pennell, Maralyn Foureur, Karen J. Campbell, Melanie Kingsland

**Affiliations:** 1 Population Health, Hunter New England Local Health District, Wallsend, NSW 2287 Australia; 2 School of Medicine and Public Health, College of Health Medicine and Wellbeing, The University of Newcastle, Callaghan, NSW 2308 Australia; 3 Hunter Medical Research Institute, New Lambton Heights, NSW 2305 Australia; 4 School of Psychological Sciences, College of Engineering, Science and Environment, University of Newcastle, Newcastle, NSW 2300 Australia; 5 Newcastle Business School, University of Newcastle, Newcastle, NSW 2300 Australia; 6 Maternity and Gynaecology, John Hunter Hospital, New Lambton Heights, NSW 2305 Australia; 7 Centre for Precision Medicine in Perinatal Health, School of Medicine and Public Health, The University of Newcastle, Callaghan, NSW 2308 Australia; 8 Nursing and Midwifery Research Centre, Hunter New England Local Health District, Newcastle, NSW 2300 Australia; 9 Centre for Research Excellence, Early Prevention of Childhood Obesity (EPOCH), Deakin University, Geelong, VIC 3220 Australia; 10 Institute for Physical Activity and Nutrition (IPAN), School of Exercise and Nutrition Sciences, Deakin University, Burwood, VIC 3125 Australia

**Keywords:** Barriers, Prioritisation, Implementation, Best–worst scaling, Antenatal care, Gestational weight gain

## Abstract

**Background:**

Antenatal clinical guidelines recommending the provision of care for gestational weight gain (GWG) are not routinely delivered by antenatal care providers (ACPs). Determining barriers to such care delivery can inform the development of targeted strategies to improve implementation. However, no previous studies have identified which barriers are most important.

**Methods:**

A best–worst scaling (BWS) survey was developed to estimate the magnitude and rank the importance of barriers to the delivery of recommended GWG care. The survey was conducted between December 2020 and November 2021 with ACPs (medical, midwifery, and Aboriginal health workers) who provided care in public maternity services within three sites in New South Wales, Australia. ACPs were asked to select which of four barriers were most and least likely to inhibit five recommended GWG care practices (assessment of GWG; advice on GWG, diet and physical activity; and referral to specialist GWG services). Rankings of barriers were determined through choice frequency analysis for ACPs at each site.

**Results:**

A total of 143 ACPs completed the survey (64.4% response rate). For each of the five recommended GWG care practices, the most important barrier across all health sites and for both midwives and medical ACPs was ‘compared to other aspects of my job, the guideline care is not a high priority.’ There was some variation in the importance of barriers between sites. Across all sites, medical staff were more likely than midwives to report ‘I forget’ as a barrier to weighing and providing weight tracking and dietary advice and ‘I don’t feel confident (providing this GWG care practice)’ as a barrier to providing referrals to a specialist GWG service.

**Conclusions:**

Best–worst scaling was a valuable method to rank the influence of barriers and to prioritise site-specific and profession-based barriers to ACP provision of guideline-recommended care for GWG. Not all barriers were equally important, and this ‘hierarchy’ differed across ACPs and sites. Implementation strategies should be developed to address the highest priority barriers, tailored to site and professional needs.

**Trial registration:**

Australian and New Zealand Clinical Trials Registry, ACTRN12621000054819 (22/01/2021). http://www.anzctr.org.au/Trial/Registration/TrialReview.aspx?id=380680&isReview=true

**Supplementary Information:**

The online version contains supplementary material available at 10.1186/s12913-025-13108-7.

## Background

Gestational weight gain (GWG) above National Academy of Medicine (NAM) recommendations [1] is a contributor to increasing population rates of caesarean birth, instrument-assisted vaginal birth, and incidence of shoulder dystocia [2]. GWG below NAM recommendations [1] has been associated with preterm birth, small for gestational age, increased risk of caesarean birth, and increased risk of infant morbidity and mortality in the first 12 months of life [2–4]. Internationally, approximately 70 per cent of pregnant women^1^ gain weight outside of the NAM recommended ranges [2, 5, 6].

Complementary to recommendations regarding GWG, pregnant women are encouraged to meet dietary [7], physical activity and sedentary behaviour guidelines [8] to support GWG within recommended ranges. However, very few pregnant women meet all dietary [9] and physical activity recommendations during pregnancy [10–12].

Public antenatal care providers (ACPs) are well-placed and trusted to provide care for GWG, dietary intake, and physical activity during pregnancy [13], with large numbers of pregnant women (e.g., 77% in Australia [14]; 80.9% in 21 European countries [15]) accessing their care. Many national pregnancy care guidelines [1, 6, 16–18], recommend that ACPs provide women with care related to GWG, dietary intake, and physical activity as part of their routine antenatal care at each appointment. These recommendations include offering to assess weight and monitor GWG; providing brief behavioural support for weight gain, dietary intake, and physical activity; and offering referral to specialist services (e.g., dietitian; health coaches) for additional support.

Internationally, there is inconsistent and low provision of guideline-recommended care for GWG. Evidence from a 2018 narrative review [19] of studies from countries including Canada, UK and Australia found that between 15%—84% of women were weighed during antenatal appointments, 9%—85% received correct GWG advice, 28%—69% received dietary advice, and 32%—46% received physical activity advice. A 2021 systematic review of 17 cross-sectional and cohort studies (*n *= 20,717) found that only 50% of ACP advice on GWG was consistent with NAM guidelines [20]. A 2024 Australian cross-sectional study involving 514 pregnant women found that 13% reported receiving a weight assessment, 30% advice on recommended weight gain, diet and physical activity, and 10% a referral to a health coach or dietitian. Less than 7% reported receiving all recommended assessment and advice care practices [21].

Understanding ACP barriers to implementing recommended GWG care can enable development of implementation strategies to support increases in care delivery. By understanding any differences in barriers between groups of ACPs (such as based on location or profession), these strategies may be further targeted. The use of determinant theories and frameworks such as the Theoretical Domains Framework (TDF) is recommended to identify barriers to implementation of guideline recommended care, by defining the structural and psychological processes that affect behavioural change [22]. Previous qualitative and quantitative studies of barriers to ACP implementation of GWG care in countries including the U.S., Canada and Australia [23–39], have identified a number of potential barriers. These include time limitations, poor or limited resources, lack of awareness of local and international GWG care guidelines, lack of training and education, costs associated with provision of GWG care, lack of referral options, gaps in communication between ACPs, funding issues, and perceptions of weight, diet and physical activity discussions being sensitive or difficult topics. These previous studies were often conducted in single locations [27, 31], and had relatively low response rates (12%−42.5%) [27, 33, 38]. No studies compared barriers between ACP groups [23, 24, 28, 30] or sites [23–27, 32, 33, 37, 38]. Only three studies used a theory-based framework [31, 33, 36] and no studies used a method to prioritise reported barriers. Prioritisation of barriers is essential to efficiently allocate resources to the most relevant implementation strategies.

Quantitative research into barriers to providing care for GWG to date have primarily used simple scaling methods such as rating and ranking systems (e.g., Likert scales) [38, 40]. There are inherent limitations in using such approaches, including results that may not be discriminating (e.g., all barriers may be rated as important), and/or difficulties in rating a large number of barriers [41, 42]. Ranking systems are limited in that while respondents may readily pick extremes, preferences for ‘in between’ options are often inaccurate [41]. Ranking also only gives an order of importance, but yields no information on the relative strength of importance and therefore does not adequately inform the distribution of resources in the development of strategies to address barriers [41].

Best–worst scaling is a maximum-difference scaling method increasingly used in health services research to identify and rank priority barriers to care, and addresses many of the limitations of simple ranking and rating approaches [42–46]. It assumes participants will seek to maximise utility when faced with a choice or decision on how to behave or act, and will choose the option that serves them best, or most aligns with their preferences. It allows participants to prioritize and directly compare the relative importance of clearly defined pre-determined attributes in a given example. It asks participants to indicate only the best and worst alternatives and thereby removes the option to select all barriers as important, and reduces the cognitive burden with ranking every single option. The repetition of best–worst choices across different scenarios allows a calculation of the order and strength of importance of barriers. By using this information, tailored strategies to address barriers may be developed, based on their likely importance.

The primary aim of this study was to use best–worst scaling to rank priority barriers of ACPs in the implementation of guideline recommended GWG care. The secondary aim was to assess whether there were differences in priority barriers based on site/location of services, and profession of ACP.

## Methods

### Design

Using a cross-sectional design, ACPs were surveyed on barriers to implementing GWG clinical guidelines using best–worst scaling. The surveys were conducted between December 2020 and November 2021.

### Setting

All public maternity services within three sectors of a Local Health District in NSW, Australia representing metropolitan/urban, inland rural/regional and coastal regional areas. These sites were chosen as a representative sample of population and service locations within the local health district. Services included midwifery group practices, midwifery clinics, specialist medical services, Aboriginal Maternal Infant Health Services (AMIHS), and multi-disciplinary teams caring for women with complex pregnancies or identified vulnerabilities. The maternity services within these three sectors provide public antenatal care to over 6000 women annually [47].

### Ethics

The study was approved by the Hunter New England Human Research Ethics Committee (16/10/19/5.15) and the University of Newcastle Human Research Ethics Committee (H-2016–0422).

### Participants eligibility

Providers of antenatal care including midwives (registered midwives, clinical midwife specialists, clinical midwife educators, clinical midwife consultants, community liaison midwives), medical practitioners (staff specialists in obstetrics, registrars, resident medical officers, general practice obstetricians and fellows), Aboriginal health practitioners, Aboriginal health workers, and student midwives and doctors were eligible to participate if they were currently employed by a participating service and had provided antenatal care within the previous 12 months. The 12-month time period enabled inclusion of any currently employed eligible staff who had work rotations to other clinical or managerial positions, which is common practice within Australian maternity care settings. People were ineligible if they did not provide direct or core antenatal care, such as managers and allied health professionals, and students rotating briefly through antenatal clinics.

### Recruitment

ACPs from participating sites were invited to complete a 10-min survey reporting their perceived barriers to providing antenatal care addressing GWG. A research assistant attended the maternity services in person to invite eligible staff to complete the surveys, for example, within education sessions and staff meetings. Sites were visited on multiple occasions to collect as many responses as possible from eligible staff and representation across all ACP groups.

### Data collection procedure and measures

The research assistant provided eligible staff with the survey, using either a paper-based *(Supplementary file: BWS Survey (GWG Care Questions)—Paper Form)* or online version using REDCap electronic data capture software [48, 49]. All paper completions were entered into the same REDCap dataset. Both versions were offered to allow for respondent preference and to overcome internet connectivity constraints. The research assistant first explained the purpose of the survey and worked through an example question. ACPs were asked about their barriers to the five specific recommended GWG care practices, that align with the guideline recommended care for GWG in routine antenatal appointments [18], which evidence suggests are poorly implemented by ACPs [30, 50–52]: (1) assessing weight at follow up appointments; (2) providing advice on recommended weight gain and how patient is tracking relative to this recommendation; (3) providing advice about healthy eating; (4) providing advice about physical activity; and (5) offering a referral to ‘Get Healthy in Pregnancy’– a government-funded telephone-based coaching service [53, 54].

Based on the model proposed by Mulbacher et al. [42], for each of these care practices, ACPs were asked to indicate which one of three presented potential barriers they considered ‘most likely to be a barrier’ to providing this care and which one would be ‘least likely to be a barrier.’ *(Supplementary file: BWS Survey (GWG Care Questions)—Paper Form).* The barrier choices (Table 2) were specific to each of the individual GWG care elements and based on previous evidence of commonly reported barriers to GWG care [30, 55], and formative results of a clinician barriers survey based on the TDF [56] undertaken by the study team. The most reported barrier determined from previous research, ‘lack of time’, was reframed as ‘compared to other aspects of my job, it isn’t a high priority’ in this survey. It was recognised that antenatal visits were time-limited appointments in which many other care practices were also expected to be provided, and hence ACPs needed to prioritise care. A total of four barrier options were arranged into four combination sets, each containing three choices.^2^ In developing the survey, a balanced incomplete block design (BIBD) was applied, such that each barrier was presented an equal number of times across the four questions (three times), and that each pair of barriers was presented an equal number of times across the four questions (two times). The order of the questions was arranged according to the Australian clinical guideline [18] recommended assess-advise-refer care pathway. The order in which the three barriers were presented was randomised so that for each question, each barrier had an equal chance of being presented first, second or third. After each survey question set, a free text section was provided where respondents could indicate anything else not listed which might be a barrier in their provision of that care practice.

### Statistical analysis

Results of the BWS quantitative survey were analysed for all respondents, and then by site, and by profession. Categories of professions for analysis were collapsed into two groups. The category of ‘midwives’ comprised registered midwives, students of midwifery, and midwifery or nursing unit managers. The ‘medical’ category comprised all roles by medical staff, and included residents, registrars, medical students, staff specialists, and obstetricians. Aboriginal health workers were not able to be included in the profession-based analysis due the small number of participants. ACP outcomes were not analysed according to years of experience, due to insufficient statistical power. Any additional free-text responses were not included in this paper’s analysis of results.

The frequency analysis, implemented by the ‘support.BWS’ statistical package in R [57], was used to analyse the survey data. A best–worst (BW) score was calculated for each respondent by subtracting the number of times the barrier was selected as ‘least likely to be a barrier’ (W) from the number of times it was selected as ‘most likely to be a barrier’ (B). In BWS surveys, the concept of ‘best’ can be considered the choice response a participant deems to be of most significance or importance, and ‘worst’ aligns with the least significant or important response (in this case, barrier). The mean and standard error (SE) of the BW scores was calculated for all respondents, and separately for each location (sites 1, 2 and 3) and profession (midwifery and medical professionals) to determine the most likely and least likely barriers for each. The larger the mean in a positive direction, the more important the barrier was determined to be, and the larger the mean in a negative direction, the less important the barrier was determined to be. Means close to zero indicated that, on average, this barrier neither clearly was, nor clearly was not, an important barrier. The means are ordered from most positive to most negative to produce a rank order of each barrier.

## Results

### Survey participation

A total of 143 out of 222 eligible ACPs completed the BWS survey (64.4% response rate). The response rates were 62.5% for Site 1: Regional/rural (35/56), 61.8% for Site 2: Regional/coastal (21/34), and 65.9% for Site 3: Metropolitan/urban (87/132). As shown in Table 1, there was less variation in ACPs surveyed at Site 2, with all being midwives. Across the sites, 48% of respondents had 10 or more years of antenatal care experience, and just over half (54%) had been in their current position for 4 years or less. Most respondents were midwives (72%).

**Table 1:** Antenatal Care Provider Characteristics

**Characteristic**	**Site 1** (n = 35) **Regional/rural**		**Site 2** (*n* = 21) **Regional/coastal**		**Site 3** (*n* = 87) **Metropolitan/urban**		**TOTAL** (*n* = 143)	
	**(n)**	**(%)**	**(n)**	**(%)**	**(n)**	**(%)**	**(n)**	**(%)**
**Years providing antenatal care**								
0–4	12	34.3	5	23.8	30	34.5	47	32.9
5–9	6	17.5	5	23.8	10	11.5	21	14.7
10 +	14	40.0	10	47.6	44	50.6	68	47.6
Not stated	3	8.6	1	4.8	3	3.4	7	4.9
**Current Position**								
Midwifery	25	71.4	19	90.5	59	67.8	103	72.0
Medical	3	8.6	0	0	11	12.6	14	9.8
Aboriginal Health Worker	1	2.9	0	0	5	5.7	6	4.2
Student Midwife	4	11.4	1	4.8	6	6.9	11	7.7
Midwifery/Nursing Manager	1	2.9	1	4.8	3	3.4	5	3.5
Staff Specialist	1	2.9	0	0	3	3.4	4	2.8
**Years in current position**								
0–4	17	48.6	12	57.1	48	55.2	77	53.8
5–9	6	17.1	1	4.8	13	14.9	20	14.0
10 +	9	25.7	7	33.3	24	27.6	40	28.0
Not stated	3	8.6	1	4.8	20	2.3	6	4.2

### Barriers to GWG Care Behaviours

**Fig. 1:**
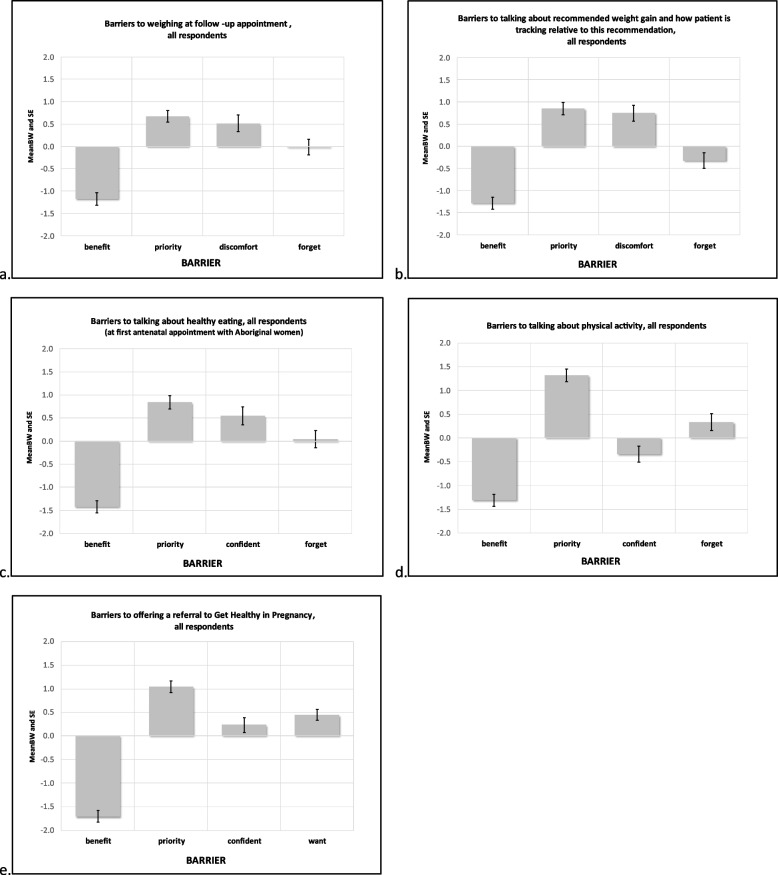
ACP barriers to provision of GWG care practices; graphing mean (and SE) BW score for barriers, all respondents. ***Legend:*** *Priority = “Compared to other aspects of my job, it isn’t a high priority”Benefit = “I don’t think it will benefit [the woman’s] health”Discomfort = “I think [the pregnant woman] may feel uncomfortable if we talk about weight gain”Forget = “I forget”Confident = “I don’t feel confident in [providing this GWG practice]”Want = “I don’t think [the pregnant woman] will want a GHiP referral”*

**Fig. 2:**
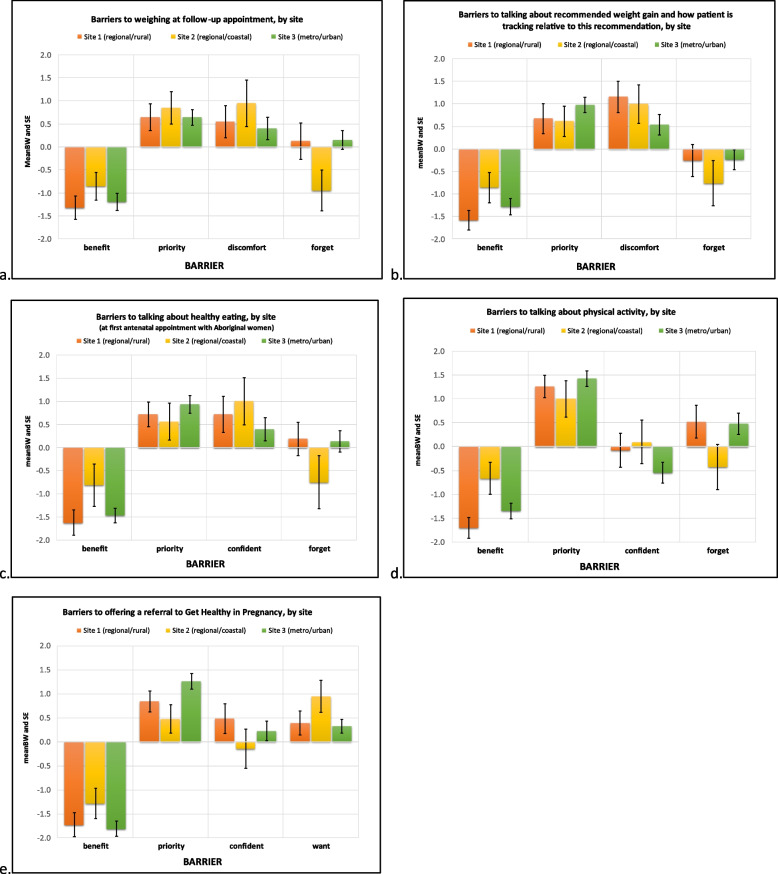
ACP barriers to provision of GWG care practices; graphing mean (and SE) BW score for barriers, by site. ***Legend:**** Priority = “Compared to other aspects of my job, it isn’t a high priority”Benefit = “I don’t think it will benefit [the woman’s] health”Discomfort = “I think [the pregnant woman] may feel uncomfortable if we talk about weight gain”Forget = “I forget”Confident = “I don’t feel confident in [providing this GWG practice]”Want = “I don’t think [the pregnant woman] will want a GHiP referral”*

**Fig. 3:**
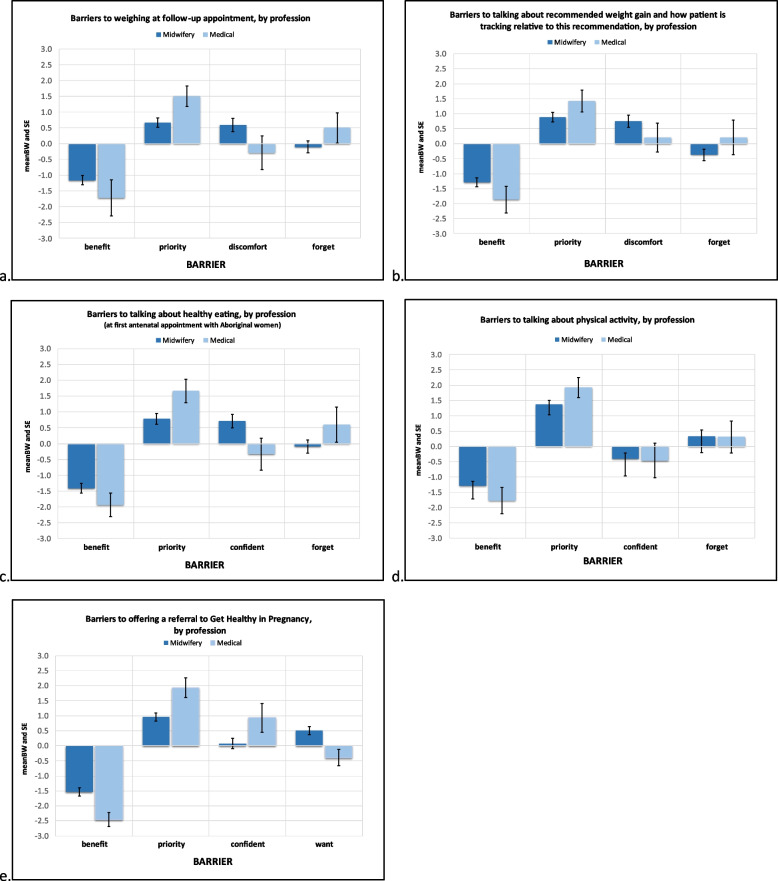
ACP barriers to provision of GWG care practices; graphing mean (and SE) BW score for barriers, by profession. ***Legend: ****Priority = “Compared to other aspects of my job, it isn’t a high priority”Benefit = “I don’t think it will benefit [the woman’s] health”Discomfort = “I think [the pregnant woman] may feel uncomfortable if we talk about weight gain”Forget = “I forget”Confident = “I don’t feel confident in [providing this GWG practice]”Want = “I don’t think [the pregnant woman] will want a GHiP referral”*

The reported mean BW scores reflect the importance and relative magnitude of each barrier for each of five GWG care practices. These means (and SEs) are presented in Fig. 1 for all respondents, Fig. 2 for site comparisons, and Fig. 3 for profession comparisons. The ranks, raw B and W counts, mean, SE and 95% CI of BW scores for all participants, each site and professional group are shown in Table 2. The rank number in Table 2 refers to the relative importance of each identified barrier’s mean BW score. The highest rank (#1) is therefore associated with the most important barrier found when calculating mean BS scores for a given care practice behaviour.

#### Weighing at follow-up appointment (Figs. 1a, 2a, 3a)

For weighing at follow-up appointments, the top two highest ranked barriers for all respondents and each site were ‘Compared to other aspects of my job, it isn’t a high priority’ and ‘I think it may make [the pregnant woman] feel uncomfortable’. ‘Compared to other aspects of my job, it isn’t a high priority’ was identified as an important barrier for both professions. For midwives ‘I think it may make [the pregnant woman] feel uncomfortable’ was also found to be an important barrier whereas for medical professionals ‘I forget’ was more important.

#### Talking about recommended weight gain and how patient is tracking relative to this recommendation (Figs. 1b, 2b, 3b)

For all respondents, Site 3 and both professions ‘Compared to other aspects of my job, it isn’t a high priority’ was the most important barrier to talking about recommended weight gain and how patient is tracking. For the remaining two sites ‘I think it may make [the pregnant woman] feel uncomfortable’ was the most important barrier, followed by ‘Compared to other aspects of my job, it isn’t a high priority’.

#### Talking about healthy eating (Figs. 1c, 2c, 3c)

When asked to consider barriers to talking about healthy eating, all respondents and participants from Sites 1 and 3 reported ‘Compared to other aspects of my job, it isn’t a high priority’ as the most important barrier, with Site 2 reporting ‘I don’t feel confident discussing anything that may be seen as judgemental with Aboriginal women’^3^ as the most important barrier. Both professional groups selected ‘Compared to other aspects of my job, it isn’t a high priority’ as the most important barrier. Additionally, for medical professionals ‘I forget’ was identified as a moderately important barrier, whereas for midwives ‘I don’t feel confident discussing anything that may be seen as judgemental with Aboriginal women’ was an important barrier.

#### Talking about physical activity (Figs. 1d, 2d, 3d)

For all respondents, and for each site and professional group ‘Compared to other aspects of my job, it isn’t a high priority’ was the most important barrier to discussing physical activity with pregnant women.

#### Offering a referral to the Get Healthy in Pregnancy (GHiP) Telephone Coaching Service (Figs. 1e, 2e, 3e)

When asked to consider barriers related to offering a referral to the ‘Get Healthy in Pregnancy’ telephone coaching service, all respondents and Sites 1 and 3 selected ‘Compared to other aspects of my job, it isn’t a high priority’ as the most important barrier, whereas Site 2 chose ‘I don’t think [the pregnant woman] will want a GHiP referral’. Both professional groups chose ‘Compared to other aspects of my job, it isn’t a high priority’ as their most important barrier. ‘I don’t feel confident in talking about the GHiP service’ was a moderately important barrier for medical professionals whereas for midwives ‘I don’t think [the pregnant woman] will want a GHiP referral’ was an important barrier.

For each of the five care practices, the least important barrier across all respondents, sites and professional groups was ‘I don’t think it will benefit [the pregnant woman’s] health’.

**Table 2:** Barriers to GWG care, ranks, best (B) and worst (W) counts, means, SEs and 95% CIs for BW scores for all respondents, and by site and profession

**1. Behaviour: Weighing at follow-up appointment**						
**Barrier/response**	**All Respondents** *n* = 138	**Site 1** **Regional/rural** *n* = 31	**Site 2** **Regional/coastal** *n* = 20	**Site 3** **Metro/urban** *n* = 87	**Midwifery** *n* = 110*	**Medical** *n* = 14*
Compared to other aspects of my job, it isn’t a high priority	Rank = 1 B = 166 W = 73 BW Scores Mean = 0.67 SE = 0.13 95% CI: 0.41, 0.94	Rank = 1 B = 35 W = 15 BW Scores Mean = 0.65 SE = 0.29 95%CI:0.05,1.24	Rank = 2 B = 29 W = 12 BW Scores Mean = 0.85 SE = 0.58 95%CI:0.11,1.58	Rank = 1 B = 102 W = 46 BW Scores Mean = 0.64 SE = 0.17 95%CI:0.31,0.98	Rank = 1 B = 133 W = 60 BW Scores Mean = 0.66 SE = 0.15 95%CI:0.37,0.96	Rank = 1 B = 23 W = 2 BW Scores Mean = 1.50 SE = 0.33 95%CI:0.79,2.21
I think Sarah may feel uncomfortable if I ask to weigh her	Rank = 2 B = 194 W = 123 BW Scores Mean = 0.51 SE = 0.19 95%CI:0.15,0.88	Rank = 2 B = 43 W = 26 BW Scores Mean = 0.55 SE = 0.35 95%CI:−0.17,1.27	Rank = 1 B = 34 W = 15 BW Scores Mean = 0.95 SE = 0.51 95%CI:−0.11,2.01	Rank = 2 B = 117 W = 82 BW Scores Mean = 0.40 SE = 0.24 95%CI:−0.08,0.88	Rank = 2 B = 162 W = 97 BW Scores Mean = 0.59 SE = 0.21 95%CI:0.17,1.01	Rank = 3 B = 11 W = 15 BW Scores Mean = −0.29 SE = 0.54 95%CI:−1.45,0.88
I forget	Rank = 3 B = 133 W = 135 BW Scores Mean = −0.01 SE = 0.17 95%CI:−0.36,0.32	Rank = 3 B = 38 W = 34 BW Scores Mean = 0.13 SE = 0.40 95%CI:−0.67,0.93	Rank = 4 B = 10 W = 29 BW Scores Mean = −0.95 SE = 0.44 95%CI:−1.88,−0.02	Rank = 3 B = 85 W = 72 BW Scores Mean = 0.15 SE = 0.21 95%CI:−0.26,0.56	Rank = 3 B = 102 W = 113 BW Scores Mean = −0.10 SE = 0.19 95%CI:−0.48,0.28	Rank = 2 B = 16 W = 9 BW Scores Mean = 0.50 SE = 0.48 95%CI:−0.53,1.53
I don’t think it will benefit Sarah’s health	Rank = 4 B = 46 W = 208 BW Scores Mean = −1.17 SE = 0.14 95%CI:−1.45,−0.90	Rank = 4 B = 5 W = 46 BW Scores Mean = −1.32 SE = 0.25 95%CI:−1.83,−0.81	Rank = 3 B = 6 W = 23 BW Scores Mean = −0.85 SE = 0.30 95%CI:−1.48,−0.22	Rank = 4 B = 35 W = 139 BW Scores Mean = −1.20 SE = 0.19 95%CI:−1.57,−0.83	Rank = 4 B = 36 W = 163 BW Scores Mean = −1.15 SE = 0.15 95%CI:−1.46,−0.85	Rank = 4 B = 6 W = 30 BW Scores Mean = −1.71 SE = 0.57 95%CI:−2.94,−0.49
**2. Behaviour: Talking about recommended weight gain and how patient is tracking relative to this recommendation**						
**Barrier/response**	**All Respondents** n = 137	**Site 1** **Regional/rural** n = 31	**Site 2** **Regional/coastal** n = 21	**Site 3** **Metro/urban** n = 85	**Midwifery** n = 111*	**Medical** n = 14*
I think Jessica may feel uncomfortable if we talk about weight gain	Rank = 2 B = 198 W = 95 BW Scores Mean = 0.75 SE = 0.17 95%CI:0.41,1.10	Rank = 1 B = 53 W = 17 BW Scores Mean BW = 1.16 SE = 0.35 95%CI:0.45,1.87	Rank = 1 B = 34 W = 13 BW Scores Mean = 1.00 SE = 0.43 95%CI:0.11,1.89	Rank = 2 B = 111 W = 65 BW Scores Mean = 0.54 SE = 0.23 95%CI:0.09,0.99	Rank = 2 B = 164 W = 80 BW Scores Mean = 0.76 SE = 0.20 95%CI:0.37,1.15	Rank = 2 B = 11 W = 8 BW Scores Mean = 0.21 SE = 0.48 95%CI:−0.83,1.26
Compared to other aspects of my job, it isn’t a high priority	Rank = 1 B = 187 W = 70 BW Scores Mean = 0.85 SE = 0.14 95%CI:0.58,1.12	Rank = 2 B = 40 W = 19 BW Scores Mean = 0.68 SE = 0.33 95%CI:−0.00,1.35	Rank = 2 B = 24 W = 11 BW Scores Mean = 0.62 SE = 0.33 95%CI:−0.87,1.32	Rank = 1 B = 123 W = 40 BW Scores Mean = 0.98 SE = 0.17 95%CI:0.64,1.32	Rank = 1 B = 151 W = 52 BW Scores Mean = 0.89 SE = 0.16 95%CI:0.58,1.20	Rank = 1 B = 26 W = 6 BW Scores Mean = 1.43 SE = 0.36 95%CI:0.65,2.20
I forget	Rank = 3 B = 120 W = 164 BW Scores Mean = −0.32 SE = 0.18 95%CI:−0.67,0.03	Rank = 3 B = 29 W = 37 BW Scores Mean = −0.26 SE = 0.36 95%CI:−0.99,0.48	Rank = 3 B = 16 W = 32 BW Scores Mean = −0.76 SE = 0.50 95%CI:−1.81,0.29	Rank = 3 B = 75 W = 95 BW Scores Mean = −0.24 SE = 0.22 95%CI:−0.68,0.21	Rank = 3 B = 93 W = 133 BW Scores Mean = −0.37 SE = 0.19 95%CI:−0.75,0.01	Rank = 2 B = 16 W = 13 BW Scores Mean = 0.21 SE = 0.58 95%CI:−1.03,1.46
I don’t think it will benefit Jessica’s health	Rank = 4 B = 36 W = 212 BW Scores Mean = −1.28 SE = 0.13 95%CI:−1.55,−1.02	Rank = 4 B = 2 W = 51 BW Scores Mean = −1.60 SE = 0.22 95%CI:−2.03,−1.13	Rank = 4 B = 7 W = 25 BW Scores Mean = −0.86 SE = 0.33 95%CI:−1.55,−0.16	Rank = 4 B = 27 W = 136 BW Scores Mean = −1.28 SE = 0.18 95%CI:−1.65,−0.92	Rank = 4 B = 30 W = 172 BW Scores Mean = −1.28 SE = 0.15 95%CI:−1.58,−0.98	Rank = 4 B = 3 W = 29 BW Scores Mean = −1.86 SE = 0.44 95%CI:−2.81,−0.90
**3. Behaviour: Talking about healthy eating**						
**Barrier/response**	**All Respondents** n = 129	**Site 1** **Regional/rural** n = 32	**Site 2** **Regional/coastal** n = 16	**Site 3** **Metro/urban** n = 81	**Midwifery** n = 102*	**Medical** n = 15*
Compared to other aspects of my job, it isn’t a high priority	Rank = 1 B = 181 W = 73 BW Scores Mean = 0.83 SE = 0.15 95%CI:0.54,1.13	Rank = 1 B = 40 W = 17 BW Scores Mean = 0.72 SE = 0.27 95%CI:0.18,1.26	Rank = 2 B = 19 W = 10 BW Scores Mean = 0.56 SE = 0.40 95%CI:−0.28,1.41	Rank = 1 B = 122 W = 46 BW Scores Mean = 0.94 SE = 0.19 95%CI:0.55,1.32	Rank = 1 B = 141 W = 61 BW Scores Mean = 0.78 SE = 0.17 95%CI:0.45,1.11	Rank = 1 B = 29 W = 4 BW Scores Mean = 1.67 SE = 0.37 95%CI:0.87,2.47
I don’t feel confident discussing anything that may be seen as judgemental with Aboriginal women	Rank = 2 B = 170 W = 99 BW Scores Mean = 0.55 SE = 0.19 95%CI:0.17,0.93	Rank = 1 B = 46 W = 23 BW Scores Mean = 0.72 SE = 0.39 95%CI:−0.08,1.51	Rank = 1 B = 24 W = 8 BW Scores Mean = 1.00 SE = 0.51 95%CI:−0.08,2.08	Rank = 2 B = 100 W = 68 BW Scores Mean = 0.40 SE = 0.25 95%CI:−0.10,0.89	Rank = 2 B = 145 W = 72 BW Scores Mean = 0.72 SE = 0.22 95%CI:0.29,1.15	Rank = 3 B = 9 W = 14 BW Scores Mean = −0.33 SE = 0.50 95%CI:−1.41,0.75
I forget	Rank = 3 B = 134 W = 129 BW Scores Mean = 0.04 SE = 0.19 95%CI:−0.33,0.41	Rank = 3 B = 34 W = 28 BW Scores Mean = 0.19 SE = 0.36 95%CI:−0.55,0.92	Rank = 3 B = 14 W = 26 BW Scores Mean = −0.75 SE = 0.57 95%CI:−1.97,0.47	Rank = 3 B = 86 W = 75 BW Scores Mean = 0.14 SE = 0.23 95%CI:−0.33,0.60	Rank = 3 B = 98 W = 107 BW Scores Mean = −0.09 SE = 0.21 95%CI:−0.50,0.32	Rank = 2 B = 20 W = 11 BW Scores Mean = 0.60 SE = 0.56 95%CI:−0.60,1.80
I don’t think it will benefit Naomi’s health	Rank = 4 B = 28 W = 212 BW Scores Mean = −1.43 SE = 0.13 95%CI:−1.69,−1.15	Rank = 4 B = 7 W = 59 BW Scores Mean = −1.63 SE = 0.27 95%CI:−2.18,−1.07	Rank = 4 B = 7 W = 20 BW Scores Mean = −0.81 SE = 0.46 95%CI:−1.79,0.16	Rank = 4 B = 14 W = 133 BW Scores Mean = −1.47 SE = 0.16 95%CI:−1.79,−1.15	Rank = 4 B = 22 W = 166 BW Scores Mean = −1.4 SE = 0.15 95%CI:−1.71,−1.11	Rank = 4 B = 2 W = 31 BW Scores Mean = −1.93 SE = 0.37 95%CI:0.31,0.98
**4. Behaviour: Talking about physical activity**						
**Barrier/response**	**All Respondents** n = 128	**Site 1** **Regional/rural** n = 27	**Site 2** **Regional/coastal** n = 21	**Site 3** **Metro/urban** n = 80	**Midwifery** n = 104*	Medical n = 13*
Compared to other aspects of my job, it isn’t a high priority	Rank = 1 B = 290 W = 50 BW Scores Mean = 1.32 SE = 0.13 95%CI:1.06,1.58	Rank = 1 B = 44 W = 10 BW Scores Mean = 1.26 SE = 0.24 95%CI:0.77,1.74	Rank = 1 B = 33 W = 12 BW Scores Mean = 1.00 SE = 0.38 95%CI:0.21,1.79	Rank = 1 B = 142 W = 28 BW Scores Mean = 1.43 SE = 0.17 95%CI:1.10,1.75	Rank = 1 B = 179 W = 37 BW Scores Mean = 1.37 SE = 0.14 95%CI:1.09,1.64	Rank = 1 B = 27 W = 2 BW Scores Mean = 1.92 SE = 0.33 95%CI:1.21,2.64
I forget	Rank = 2 B = 157 W = 114 BW Scores Mean = 0.34 SE = 0.18 95%CI:−0.02,0.69	Rank = 2 B = 33 W = 19 BW Scores Mean = 0.52 SE = 0.34 95%CI:−0.19,1.22	Rank = 3 B = 19 W = 28 BW Scores Mean = −0.43 SE = 0.48 95%CI:−1.42,0.56	Rank = 2 B = 105 W = 67 BW Scores Mean = 0.48 SE = 0.23 95%CI:0.03,0.92	Rank = 2 B = 129 W = 95 BW Scores Mean = 0.33 SE = 0.20 95%CI:−0.07,0.73	Rank = 2 B = 15 W = 11 BW Scores Mean = 0.31 SE = 0.52 95%CI:−0.83,1.45
I don’t feel confident discussing physical activity in pregnancy	Rank = 3 B = 98 W = 142 BW Scores Mean = −0.34 SE = 0.17 95%CI:−0.68,−0.00	Rank = 3 B = 26 W = 28 BW Scores Mean = −0.07 SE = 0.36 95%CI:−0.81,0.66	Rank = 2 B = 21 W = 19 BW Scores Mean = 0.10 SE = 0.46 95%CI:−0.86,1.05	Rank = 3 B = 51 W = 95 BW Scores Mean = −0.55 SE = 0.22 95%CI:−0.98,−0.12	Rank = 3 B = 76 W = 118 BW Scores Mean = −0.40 SE = 0.19 95%CI:−0.77,−0.03	Rank = 3 B = 8 W = 14 BW Scores Mean = −0.46 SE = 0.56 95%CI:−1.69,0.76
I don’t think it will benefit Courtney’s health	Rank = 4 B = 27 W = 195 BW Scores Mean = −1.31 SE = 0.13 95%CI:−1.57,−1.06	Rank = 4 B = 2 W = 48 BW Scores Mean = −1.70 SE = 0.22 95%CI:−2.15,−1.25	Rank = 4 B = 6 W = 20 BW Scores Mean = −0.67 SE = 0.33 95%CI:−1.36,0.03	Rank = 4 B = 19 W = 127 BW Scores Mean = −1.35 SE = 0.17 95%CI:−1.68,−1.02	Rank = 4 B = 23 W = 157 BW Scores Mean = −1.29 SE = 0.14 95%CI:−1.57,−1.01	Rank = 4 B = 1 W = 24 BW Scores Mean = −1.77 SE = 0.43 95%CI:−2.70,−0.84
** 5. Behaviour: Offering a Referral to Get Healthy in Pregnancy (GHiP)**						
**Barrier/response**	**All Respondents** n = 134	**Site 1** **Regional/rural** n = 33	**Site 2** **Regional/coastal** n = 21	**Site 3** **Metro/urban** n = 80	**Midwifery** n = 108*	**Medical** n = 15*
Compared to other aspects of my job, it isn’t a high priority	Rank = 1 B = 203 W = 64 BW Scores Mean = 1.04 SE = 0.12 95%CI:0.80,1.28	Rank = 1 B = 44 W = 16 BW Scores Mean = 0.85 SE = 0.22 95%CI:0.40,1.29	Rank = 2 B = 24 W = 14 BW Scores Mean = 0.48 SE = 0.30 95%CI:−0.14,1.10	Rank = 1 B = 135 W = 34 BW Scores Mean = 1.26 SE = 0.16 95%CI:0.95,1.58	Rank = 1 B = 158 W = 55 BW Scores Mean = 0.95 SE = 0.13 95%CI:0.69,1.22	Rank = 1 B = 32 W = 3 BW Scores Mean = 1.93 SE = 0.33 95%CI:1.22,2.64
I don’t think Rebecca will want a GHiP referral	Rank = 2 B = 128 W = 69 BW Scores Mean = 0.44 SE = 0.12 95%CI:0.21,0.67	Rank = 3 B = 34 W = 21 BW Scores Mean = 0.39 SE = 0.25 95%CI:−0.12,0.91	Rank = 1 B = 28 W = 8 BW Scores Mean = 0.95 SE = 0.33 95%CI:0.25,1.65	Rank = 2 B = 66 W = 40 BW Scores Mean = 0.33 SE = 0.14 95%CI:0.04,0.61	Rank = 2 B = 108 W = 53 BW Scores Mean = 0.51 SE = 0.13 95%CI:0.25,0.77	Rank = 3 B = 7 W = 13 BW Scores Mean = −0.40 SE = 0.27 95%CI:−0.98,0.18
I don’t feel confident in talking about the GHiP service	Rank = 3 B = 133 W = 102 BW Scores Mean = 0.23 SE = 0.16 95%CI:−0.08,0.54	Rank = 2 B = 34 W = 18 BW Scores Mean = 0.48 SE = 0.31 95%CI:−0.15,1.12	Rank = 3 B = 16 W = 19 BW Scores Mean = −0.14 SE = 0.41 95%CI:−1.00,0.71	Rank = 3 B = 83 W = 65 BW Scores Mean = 0.23 SE = 0.20 95%CI:−0.18,0.63	Rank = 3 B = 100 W = 92 BW Scores Mean = 0.07 SE = 0.18 95%CI:−0.28,0.42	Rank = 2 B = 19 W = 5 BW Scores Mean = 0.93 SE = 0.48 95%CI:−0.10,1.97
I don’t think it will benefit Rebecca’s health	Rank = 4 B = 27 W = 256 BW Scores Mean = −1.71 SE = 0.12 95%CI:−1.95,−1.47	Rank = 4 B = 7 W = 64 BW Scores Mean = −1.73 SE = 0.25 95%CI:−2.24,−1.22	Rank = 4 B = 5 W = 32 BW Scores Mean = −1.29 SE = 0.32 95%CI:−1.95,−0.62	Rank = 4 B = 15 W = 160 BW Scores Mean = −1.81 SE = 0.16 95%CI:−2.13,−1.50	Rank = 4 B = 26 W = 192 BW Scores Mean = −1.54 SE = 0.14 95%CI:−1.81,−1.26	Rank = 4 B = 0 W = 37 BW Scores Mean = −2.47 SE = 0.24 95%CI:−2.97,−1.96

^*^The sum of respondent numbers across the three sites is equal to the total (all respondents) number. A small number of ACPs fell into the ‘other’ category and were not analysed in professional groups as they were too heterogeneous

## Discussion

The primary aim of this study was to prioritise barriers to the implementation of clinical guidelines for GWG in pregnancy, and secondarily to assess whether there were differences in the prioritisation of barriers based on site/location of services, and profession of ACPs. In a novel application, we used best–worst scaling to prioritise barriers to care. We found across all respondents that the most important barrier for all five practices of recommended GWG care was ‘Compared to other aspects of my job, it isn’t a high priority’. Other barriers related to lacking confidence in completing the care practice, concerns for the pregnant woman’s comfort level in discussing topics such as weight and dietary intake, and forgetting to complete the care practice, differed in priority based on site and ACP profession. Medical ACPs selected ‘Compared to other aspects of my job, it isn’t a high priority’ more than twice as often as other barriers, and midwives selected barriers reflecting a concern for patient discomfort and their own confidence more frequently. ‘I don’t think it will benefit [the pregnant woman’s] health’ was not identified as an important barrier for any of the care practices.

Our results add further evidence to support the findings of previous studies [23–39] that have identified barriers to providing care for GWG such as lack of confidence; concerns regarding patient sensitivity in discussing these topics; considering GWG, diet and physical activity care a low priority in antenatal care; and poor or limited resources. This is the first study, however, which extends this existing knowledge base by prioritising barriers. To enable the development of implementation strategies which support increases in the provision of recommended antenatal care for GWG, dietary intake and physical activity, the barriers prioritised through these BWS surveys should be mapped to evidence-based implementation strategies and behaviour change techniques [58–61]. For example, the most important barrier for all elements of GWG care, ‘Compared to other aspects of my job, it isn’t a high priority’, could be addressed through the provision of educational sessions that include information about health consequences from credible sources or experts, to increase the perceived importance of these care practices [62–64]. These educational sessions could also include demonstrations of how these care practices could fit into existing appointment times, and practice/rehearsal from staff in training sessions [58, 65, 66]. Restructuring the physical environment could also be used to address this barrier through reviewing the current content of appointments to determine if any care could be delivered through alternative modalities, such as assessments undertaken through pre-appointment questionnaires, freeing up appointment time for patient discussions [67–70].

Site-based differences in priority barriers to GWG care may be related to the relative size, location and antenatal services offered. When comparing site-based responses, the larger regional and metropolitan sites (1 and 3) demonstrated very similarly prioritised barriers. Respondents from the smallest site, in a regional township, more often selected barriers around the perceived psychological comfort level of pregnant women when having discussions about weight, diet, and physical activity, as well as a lack of confidence in delivering culturally appropriate care to Aboriginal pregnant women. Strategies including persuasive education that is delivered by credible sources and aims to increase confidence in delivering culturally appropriate care, could address such barriers. In the broad literature, implementation strategies which are tailored to contextual needs have been found to improve practices [60, 71, 72], however, studies assessing the effectiveness of implementation strategies to address site-based barriers are limited [73]. There is a need to test such an approach to determine whether it is effective and warranted.

Analysis of the differences between barriers reported by midwifery and medical professionals showed more variation in barrier priorities than site-based differences. For all GWG care practices, medical professions reported ‘Compared to other aspects of my job, it isn’t a high priority’ as a barrier that was more than twice as important than any other barrier. Midwives, however, also identified barriers related to potential discomfort of the topic (e.g., weight tracking, diet) or their own levels of confidence as important. One previous study that assessed profession-based differences in barriers to GWG care [37], reported lack of training as a barrier experienced more by medical staff (obstetrician/gynaecologists) than midwives or nurse practitioners. Another study similarly reported that medical ACPs viewed lack of medical school training in dietetics as a barrier [36]. Given these differences in priority barriers by ACP profession, a tailored selection of evidence-based implementation strategies for midwives and medical ACPs is likely needed to support improvements in GWG care delivery.

While this study has prioritised barriers to care provision for GWG in antenatal care, there is a need to test the effectiveness of those implementation strategies and behaviour change techniques that theoretical frameworks suggest should address these prioritised barriers. Any intervention that is tested should be appropriately co-designed with pregnant women to ensure their preferences and acceptability regarding the receipt of antenatal care practices are understood and included [21, 74–77]. Currently there have been limited controlled trials conducted to test such strategies to improve antenatal care for GWG [59, 78–80], with only one using theoretically informed strategy design [78]. A controlled implementation trial assessing the effectiveness of strategies developed based on a clear understanding of priority barriers is needed to further this evidence base.

### Study limitations and strengths

This study was conducted across three separate maternity service sites which allowed sampling of metropolitan and regional/rural ACPs and yielded a strong response rate (> 60%) across all sites. However, our study was conducted in one health district in one state of Australia and results may not be replicable or generalisable to other locations due to local contextual differences. Analysis of the study results did not consider years of experience of the ACP due to relatively small response numbers yielding insufficient study power; future studies using larger sample sizes could investigate this further. The timing of our surveys also coincided with the COVID-19 pandemic response (December 2020—November 2021), which affected maternity services operations and staffing levels, and hence this could have affected our findings, particularly regarding higher priority maternity care. Each participant received the barrier questions in the same order, which may have introduced question order (or order-effects) bias. For example, respondents may have sought to provide answers consistent with their prior responses, rather than treating each question independently, or they may have become fatigued as the questions progressed and opted for short-cut strategies resulting in attribute dominance. The minimal attrition rate across the questions and the generally consistent, but still variable, pattern of barrier ranks across the questions suggest that the risks of these biases are low. Lastly, the nature of the BWS method limits response options, unlike open-response qualitative studies. By forcing or limiting choices, the study design may have excluded other valid barrier options as it relies on previously determined commonly understood barriers as the basis for the options offered. Qualitative studies exploring these would also be beneficial to confirm our findings.

## Conclusions

Not being a high priority compared to other aspects of antenatal care was found to be the most salient barrier to the implementation of all GWG care practices. Site and profession-based differences were also found, including medical staff forgetting to provide care and staff from the smallest site feeling less confident to deliver care due to concerns of negative patient reactions. The BWS survey method was used to determine not only the ranking of commonly identified barriers to care, but the relative magnitude of these when compared to other barriers and provided the ability to assess differences in priority barriers based on site and profession. These results should be used to inform the development of highly tailored interventions to improve the delivery of antenatal care for GWG.

## Supplementary Information

Supplementary Material 1

## Data Availability

The datasets used and analysed during this study are available from the corresponding author on reasonable request.

## Abbreviations

GWG: Gestational weight gain
ACP: Antenatal care provider
BWS: Best-worst scaling
TDF: Theoretical domains framework

## Footnotes

1 This paper uses the term 'pregnant woman/women' throughout; however, it is recognised that people who do not identify as women may be pregnant and included in these descriptions.

2 All participants completed all four choices, which were arranged in the same order - within and across the choices, because of the use of printed surveys.

3 The barrier ‘I don’t feel confident discussing anything that may be seen as judgemental with Aboriginal women’ was included as the question was based on a scenario where the pregnant woman identified as Aboriginal.
