# Supplementary material for: Implementing clinical guidelines for gestational weight gain care: a novel application of best–worst scaling to prioritise barriers
Source: BMC Health Serv Res. 2025 Jul 29;25:997. doi: 10.1186/s12913-025-13108-7 (PMC12308902; doi:10.1186/s12913-025-13108-7)
Supplement: Supplementary file 1 — Supplementary Material 1 [file 12913_2025_13108_MOESM1_ESM.pdf]

Health
Hunter New England
Local Health District

THE UNIVERSITY OF NEWCASTLE AUSTRALIA

# STAFF SURVEY

## Antenatal care addressing alcohol consumption, gestational weight gain, and smoking in pregnancy

**Instructions:**

- Thank you for taking the time to complete this survey.
- There are no right or wrong answers for this survey – some questions may seem like there is no suitable answer for you, but please respond with the answer that is closest to what you feel.
- We understand that the care you deliver depends on the specific needs of the woman, however, please respond generally in terms of antenatal care you provide.
- The style of the questions means the response options repeat. This is so we get the most accurate idea of how you feel about the situations presented and your specific priorities for antenatal care for the women you care for.

The questions and response options have been informed by feedback we have previously received from HNE maternity services.

***First, we would like to ask some information about yourself and your role. This information will be treated as confidential and stored separate to your responses.***

| | |
|---|---|
| Staff number: | |
| First name: | |
| Last name: | |
| Unit/service: | |
| What is your position (e.g. staff specialist, registered midwife, CME, AHW etc.)? | |
| How many years have you worked in your current position? | |
| For how many years have you been providing antenatal care? | |

**Example question:**

**The next page shows an example question and completed responses.**

***Imagine you are at your regular check-up appointment at the dentist and they recommend that you floss your teeth twice a day.***

For each table below, the responses have been selected for what is **most likely to be a barrier** and **least likely to be a barrier** that might prevent someone from **flossing twice a day**.

**Example completed response options**

| Most likely to be a barrier | | Least likely to be a barrier |
|---|---|---|
| x | I forget | |
| | I don't believe it will benefit my health | |
| | I don't know how | x |

| Most likely to be a barrier | | Least likely to be a barrier |
|---|---|---|
| x | I forget | |
| | I don't believe it will benefit my health | |
| | I don't have any floss at home | x |

| Most likely to be a barrier | | Least likely to be a barrier |
|---|---|---|
| x | I don't believe it will benefit my health | |
| | I don't know how | |
| | I don't have any floss at home | x |

| Most likely to be a barrier | | Least likely to be a barrier |
|---|---|---|
| x | I forget | |
| | I don't know how | |
| | I don't have any floss at home | x |

**Please turn to the next page to begin the survey**

- Please read the following scenario and respond to the questions in a way that **reflects your usual practice**.
- The statements and response options have been carefully devised in order to explore your barriers in providing antenatal care for gestational weight gain, alcohol use and smoking. **These questions are deliberately repetitious, and their subtle differences are an important part of the evaluation.**
- You may not perceive one/more of these responses as a barrier at all. Please select the response which is of **most concern** as ***'most likely to be a barrier'*** and the response that is of **least concern** as ***'least likely to be a barrier'.***

***Imagine that you are at a follow up antenatal appointment with Sarah. As part of routine care it is recommended that you measure her weight.***

Please indicate which response below is **most likely to be a barrier** and which is **least likely to be a barrier** in preventing you from **weighing Sarah** at this follow up appointment.

| Most likely to be a barrier | | Least likely to be a barrier |
|---|---|---|
| | I don't think it will benefit Sarah's health | |
| | Compared to other aspects of my job, it isn't a high priority | |
| | I think Sarah may feel uncomfortable if I ask to weigh her | |

| Most likely to be a barrier | | Least likely to be a barrier |
|---|---|---|
| | I think Sarah may feel uncomfortable if I ask to weigh her | |
| | I don't think it will benefit Sarah's health | |
| | I forget | |

| Most likely to be a barrier | | Least likely to be a barrier |
|---|---|---|
| | Compared to other aspects of my job, it isn't a high priority | |
| | I forget | |
| | I don't think it will benefit Sarah's health | |

| Most likely to be a barrier | | Least likely to be a barrier |
|---|---|---|
| | I think Sarah may feel uncomfortable if I ask to weigh her | |
| | Compared to other aspects of my job, it isn't a high priority | |
| | I forget | |

Is there anything not listed that would prevent you from **weighing Sarah** at a follow up appointment?

______________________________

______________________________

______________________________

***Imagine that you are in the first antenatal appointment with Jessica. As part of routine care, it is recommended that you have a conversation about recommended weight gain and how her weight gain is tracking in her pregnancy.***

Please indicate which response below is **most likely to be a barrier** and which is **least likely to be a barrier** in preventing you from talking to Jessica about her **recommended weight gain** and how she is tracking relative to this recommendation.

| Most likely to be a barrier | | Least likely to be a barrier |
|---|---|---|
| | I don't think it will benefit Jessica's health | |
| | Compared to other aspects of my job, it isn't a high priority | |
| | I think Jessica may feel uncomfortable if we talk about weight gain | |

| Most likely to be a barrier | | Least likely to be a barrier |
|---|---|---|
| | I don't think it will benefit Jessica's health | |
| | I think Jessica may feel uncomfortable if we talk about weight gain | |
| | I forget | |

| Most likely to be a barrier | | Least likely to be a barrier |
|---|---|---|
| | I forget | |
| | I don't think it will benefit Jessica's health | |
| | Compared to other aspects of my job, it isn't a high priority | |

| Most likely to be a barrier | | Least likely to be a barrier |
|---|---|---|
| | I forget | |
| | Compared to other aspects of my job, it isn't a high priority | |
| | I think Jessica may feel uncomfortable if we talk about weight gain | |

Is there anything not listed that would prevent you from **talking about weight gain and weight gain tracking** with Jessica?

___

___

___

***Imagine that you are in the first antenatal appointment with Naomi who is an Aboriginal woman. As part of routine care, it is recommended that you have a conversation about healthy eating in pregnancy.***

Please indicate which response below is **most likely to be a barrier** and which is **least likely to be a barrier** in preventing you from talking to Naomi about **healthy eating** at this appointment?

| Most likely to be a barrier | | Least likely to be a barrier |
|---|---|---|
| | Compared to other aspects of my job, it isn’t a high priority | |
| | I don’t feel confident discussing anything that may be seen as judgemental with Aboriginal women | |
| | I don’t think it will benefit Naomi’s health | |

| Most likely to be a barrier | | Least likely to be a barrier |
|---|---|---|
| | I don’t feel confident discussing anything that may be seen as judgemental with Aboriginal women | |
| | I forget | |
| | I don’t think it will benefit Naomi’s health | |

| Most likely to be a barrier | | Least likely to be a barrier |
|---|---|---|
| | I don’t think it will benefit Naomi’s health | |
| | Compared to other aspects of my job, it isn’t a high priority | |
| | I forget | |

| Most likely to be a barrier | | Least likely to be a barrier |
|---|---|---|
| | I forget | |
| | I don’t feel confident discussing anything that may be seen as judgemental with Aboriginal women | |
| | Compared to other aspects of my job, it isn’t a high priority | |

Is there anything not listed that would prevent you from talking about **healthy eating** with Naomi?

______________________________________________________________________

______________________________________________________________________

______________________________________________________________________

***Imagine that you are in an antenatal appointment with Courtney who is a 34 year old woman. As part of routine care it is recommended that you have a conversation about physical activity in pregnancy.***

Please indicate which response below is **most likely to be a barrier** and which is **least likely to be a barrier** in preventing you from talking to Courtney about **physical activity** at this appointment?

| Most likely to be a barrier | | Least likely to be a barrier |
|---|---|---|
| | I don't feel confident in discussing physical activity in pregnancy | |
| | Compared to other aspects of my job, it isn't a high priority | |
| | I don't think it will benefit Courtney's health | |

| Most likely to be a barrier | | Least likely to be a barrier |
|---|---|---|
| | I forget | |
| | I don't think it will benefit Courtney's health | |
| | I don't feel confident in discussing physical activity in pregnancy | |

| Most likely to be a barrier | | Least likely to be a barrier |
|---|---|---|
| | I don't think it will benefit Courtney's health | |
| | I forget | |
| | Compared to other aspects of my job, it isn't a high priority | |

| Most likely to be a barrier | | Least likely to be a barrier |
|---|---|---|
| | I don't feel confident in discussing physical activity in pregnancy | |
| | Compared to other aspects of my job, it isn't a high priority | |
| | I forget | |

Is there anything not listed that would prevent you from talking about the benefits of **physical activity** with Courtney?

____________________________________________________________

____________________________________________________________

____________________________________________________________

***Imagine that you are in an early antenatal appointment with Rebecca. As part of routine care, it is recommended that you offer her a referral to the Get Healthy in Pregnancy (GHiP) Telephone Service.***

Please indicate which response below is **most likely to be a barrier** and which is **least likely to be a barrier** in preventing you from offering Rebecca a **referral to GHiP Telephone Service**?

| Most likely to be a barrier | | Least likely to be a barrier |
|---|---|---|
| | I don't feel confident in talking about the GHiP Service | |
| | I don't think Rebecca will want a GHiP referral | |
| | I don't think it will benefit Rebecca's health | |

| Most likely to be a barrier | | Least likely to be a barrier |
|---|---|---|
| | Compared to other aspects of my job, it isn't a high priority | |
| | I don't think it will benefit Rebecca's health | |
| | I don't think Rebecca will want a GHiP referral | |

| Most likely to be a barrier | | Least likely to be a barrier |
|---|---|---|
| | I don't think it will benefit Rebecca's health | |
| | I don't feel confident in talking about the GHiP Service | |
| | Compared to other aspects of my job, it isn't a high priority | |

| Most likely to be a barrier | | Least likely to be a barrier |
|---|---|---|
| | I don't think Rebecca will want a GHiP referral | |
| | Compared to other aspects of my job, it isn't a high priority | |
| | I don't feel confident in talking about the GHiP Service | |

Is there anything not listed that would prevent you from offering Rebecca a **referral to the Get Healthy in Pregnancy (GHiP) Telephone Service**?

________________________________________

________________________________________

________________________________________

**Do you have any other feedback or comments?**

***Thank you for taking the time to complete this survey.***
